# Supplementary material for: The Veterinary Anti-Parasitic Selamectin Is a Novel Inhibitor of the Mycobacterium tuberculosis DprE1 Enzyme
Source: Int J Mol Sci. 2022 Jan 11;23(2):771. doi: 10.3390/ijms23020771 (PMC8776228; doi:10.3390/ijms23020771)
Supplement: Supplementary file 1 [file ijms-23-00771-s001.zip › ijms-1477253-supplementary.pdf]

SUPPORTING INFORMATION

The veterinary anti-parasitic selamectin is a novel inhibitor of the *Mycobacterium tuberculosis* DprE1 enzyme

Ezquerra-Aznárez, J.M. *et al.*

Table S1. Oligonucleotides used for DprE1 mutagenesis

Name	Sequence
DprE1 C394A For	CGATCCCGGGCTGGAACGTGGCCGTGGACTTCCCGATCAAGG
DprE1 C394A Rev	CCTTGATCGGGAAGTCCACGGCCACGTTCCAGCCCGGGATCG
DprE1 C394G For	CGATCCCGGGCTGGAACGTGGGCGTGGACTTCCCGATCAAG
DprE1 C394G Rev	CTTGATCGGGAAGTCCACGCCCACGTTCCAGCCCGGGATCG
DprE1 C394S For	GATCCCGGGCTGGAACGTGTCCGTGGACTTCCCGATCAAGG
DprE1 C394S Rev	CCTTGATCGGGAAGTCCACGGACACGTTCCAGCCCGGGATC
DprE1 L282F For	CGCCGCAACTGCTCACGTTTCCGGACATCT
DprE1 L282F Rev	AGATGTCCGGAAACGTGAGCAGTTGCGGCG
DprE1 L282V For	CGCCGCAACTGCTCACGGTGCCGGACATCT
DprE1 L282V Rev	AGATGTCCGGCACCGTGAGCAGTTGCGGCG

Table S2. Oligonucleotides used for *M. smegmatis* recombineering. Point mutations introduced with mutagenic oligos are highlighted

Name	Sequence
rpsL+	GCGACCTTCCGGAGCGCCGAGTTCGGCTTCCTCGGAGTGGTGGTGTAAACGCGCGTGCACA
L282F	AGCTGCAGAAGGATCCACTGAAATTCGATGCGCCGCAACTGCTCACGTTTCCGGACATCTTCCCGAACGGCCTGGCCAACAAGTTCACGTTCATGCCGAT
L282V	AGCTGCAGAAGGATCCACTGAAATTCGATGCGCCGCAACTGCTCACGGTTCCGGACATCTTCCCGAACGGCCTGGCCAACAAGTTCACGTTCATGCCGA
dprE1-seq-F	GTGAGCCTGGACCAGTTGATGAAAGC
dprE1-seq-R	TACAGCCTGCCACCGAACTCC

```
Mab         MARASGLCHRPGDCDSHQFHRPARGDLPNPLSRYAQLPMTTTPKSELPLTPRALTGFGRTA    60
Mav         ----------------------------------------MSSTDPLITPARLTGFGRTA    20
Msm         ------------------------------MGAV--PSLTMSTTEFPTTTKRLMGWGRTA    28
Mtb         ----------------------------------------MLSVGATTTATRLTGWGRTA    20
Mbo         ----------------------------------------MLSVGATTTATRLTGWGRTA    20
Mka         ----------------------------------------MSSNASSTTPTRLTGWGRTA    20
                                                      .     *   * *:****

Mab         PTVAHVLSTPDVDVIAEAVRQVADASSHSPAHLRRGIVARGLGRSYGDHACNGGGIVVDM    120
Mav         PSVAQVLRTRDPEVIAKAVARVADSGH----SKGRGVIARGLGRSYGDNAQNGGGLVIDM    76
Msm         PTVASVLSTSDPEVIVRAVTRAAEE-------GGRGVIARGLGRSYGDNAQNGGGLVIDM    81
Mtb         PSVANVLRTPDAEMIVKAVARVAESG------GGRGAIARGLGRSYGDNAQNGGGLVIDM    74
Mbo         PSVANVLRTPDAEMIVKAVARVAESG------GGRGAIARGLGRSYGDNAQNGGGLVIDM    74
Mka         PSVADVLRTPDPEVIAKAVARAAES-------GARGVIARGLGRSYGDNAQNGGGLVIDM    73
            *:** ** * * ::*..** :.*:          ** :**********:* ****:*:**

Mab         TPLKRVHSISAETAVADVDAGVSLDQLMKAALPFGLWVPVLPGTRQVTVGGAIGSDIHGK    180
Mav         TGLNRIHSISADTRLVDVDAGVSLDQLMKAALPFGLWVPVLPGTRQVTVGGAIACDIHGK    136
Msm         PALNRIHSIDSGTRLVDVDAGVSLDQLMKAALPHGLWVPVLPGTRQVTVGGAIGCDIHGK    141
Mtb         TPLNTIHSIDADTKLVDIDAGVNLDQLMKAALPFGLWVPVLPGTRQVTVGGAIACDIHGK    134
Mbo         TPLNTIHSIDADTKLVDIDAGVNLDQLMKAALPFGLWVPVLPGTRQVTVGGAIACDIHGK    134
Mka         SGLNNIHSISADTKLADVDAGVNLDQLMKAALPFGLWVPVLPGTRQVTVGGAIACDIHGK    133
              *: :***.: * :.*:****.**********.*******************..*****

Mab         NHHSAGSFGNHVLSMDLLMADGEVHTITPDGP---ASELFWATVGGNGLTGIVVRARIAM    237
Mav         NHHSAGSFGNHVRSMELLMADGTVRTITPDGPDASDAELFWATVGGNGLTGIVLRATIAM    196
Msm         NHHSAGSFGNHVRSMELLTANGEVRHLTPAGP---DSDLFWATVGGNGLTGIILRATIEM    198
Mtb         NHHSAGSFGNHVRSMDLLTADGEIRHLTPTGE---DAELFWATVGGNGLTGIIMRATIEM    191
Mbo         NHHSAGSFGNHVRSMDLLTADGEIRHLTPTGE---DAELFWATVGGNGLTGIIMRATIEM    191
Mka         NHHSAGSFGNHVRSMDLLLANGEVRRLSPDGD---EAELFWATVGGNGLTGIILRATIEM    190
            ************ **:** *:* :: ::* *     ::**************::** * *

Mab         TRTETAYFIADGVATRDLDETIAVHQDGTEDNYTYSSAWFDLINPPPKLGRAAVSRGSLA    297
Mav         TPTETAYFIADGVATKDLDETVAVHLDGSEADYTYSSAWFDLISPPPKLGRAAVSRGSLA    256
Msm         TPTETAYFIADGDVTGSLDETIAFHSDGSEANYTYSSAWFDAISKPPKLGRAAISRGSLA    258
Mtb         TPTSTAYFIADGDVTASLDETIALHSDGSEARYTYSSAWFDAISAPPKLGRAAVSRGRLA    251
Mbo         TPTSTAYFIADGDVTASLDETIALHSDGSEARYTYSSAWFDAISAPPKLGRAAVSRGRLA    251
Mka         TPTETAYFIADGDVTATLDETIALHSDGSEADYTYSSAWFDAISAPPKLGRAAISRGSLA    250
            * *.******** .*  ****:*.* **:*  ********* *. ********:*** **
```

```
Mab      KLDQLPPKLAKDPLKFSAPQLPGLPDLFPVNFMIKPSLMAIGEAFYRMSGNYQGKIVNLT    357
Mav      RLDQLPKKLAKNPLKFDAPQLLTVPDVFPVSAMNKLSFMAIGEVYYRLGGTYTGKVMNLS    316
Msm      KLDQLPSKLQKDPLKFDAPQLLTLPDIFPNGLANKFTFMPIGELWYRKSGTYRNKVQNLT    318
Mtb      TVEQLPAKLRSEPLKFDAPQLLTLPDVFPNGLANKYTFGPIGELWYRKSGTYRGKVQNLT    311
Mbo      TVEQLPAKLRSEPLKFDAPQLLTLPDVFPNGLANKYTFGPIGELWYRKSGTYRGKVQNLT    311
Mka      RLEQLPTKLQRNPLKFDAPQLLTFPDVFPNGLANKYTFGPIGELWYRKSGTYRGKIQNLT    310
          ::*** **  :****.****  .**:** .   * ::  *** :** .*.* .*: **:

Mab      QFYHMLDITTGWQYAYGPAGFAQHQFLVPPDALEEFKGIIRWIQTQGQYSALNVFKLFGP    417
Mav      QFYHMLDLVSGWNNAYGPRGFAQHQFLVPPDAMDEFKAIIRWIQTRGHYSALNVFKLFGP    376
Msm      QFYHPLDMFGEWNRAYGSAGFLQYQFVVPTEAVEEFKSIIVDIQRSGHYSFLNVFKLFGP    378
Mtb      QFYHPLDMFGEWNRAYGPAGFLQYQFVIPTEAVDEFKKIIGVIQASGHYSFLNVFKLFGP    371
Mbo      QFYHPLDMFGEWNRAYGPAGFLQYQFVIPTEAVDEFKKIIGVIQASGHYSFLNVFKLFGP    371
Mka      QFYHPLDMFGEWNRAYGPAGFLQYQFVIPTEAVDEFKKIIRDIQASGHYSFLNVFKLFGP    370
         **** **:   *: ***  ** *:**::* :*::*** **  **  *:** *********

Mab      GNKAPLSFPMKGWNVAMDFPNKPGVNEFLNELDNRAMEFGGRVYTAKDSRVSAEKFHRMY    477
Mav      GNRAPLSFPMAGWNVAMDFPNKPGVNEFLNELDRRVLQFGGRVYTAKDSRTNAETFHAMY    436
Msm      GNQAPLSFPIPGWNVCVDFPIKAGLHEFVTELDRRVLEFGGRLYTAKDSRTTAETFHAMY    438
Mtb      RNQAPLSFPIPGWNICVDFPIKDGLGKFVSELDRRVLEFGGRLYTAKDSRTTAETFHAMY    431
Mbo      RNQAPLSFPIPGWNICVDFPIKDGLGKFVSELDRRVLEFGGRLYTAKDSRTTAETFHAMY    431
Mka      GNRAPLSFPIPGWNICVDFPIKAGLNEFVSELDRRVLEFGGRLYTAKDSRTTAETFHAMY    430
          *:******: ***:.:*** * *: :*:.***.*.::****:*******..**.** **

Mab      PRVDEWIATRRKADPHGVFASDMARRLELL    507
Mav      PRIDEWIAVRRKVDPTGVFASDMARRLELL    466
Msm      PRIDEWIRIRRSVDPDGVFASDMARRLQLL    468
Mtb      PRVDEWISVRRKVDPLRVFASDMARRLELL    461
Mbo      PRVDEWISVRRKVDPLRVFASDMARRLELL    461
Mka      PRIDEWIAVRRKVDPLRVFASDMARRLELL    460
         **:****  **..**  **********:**
```

Figure S1. Sequence alignment of mycobacterial DprE1. Residues predicted to be relevant for selamectin binding to DprE1 are highlighted in yellow (Leu275 is shown in red) *Mab: Mycobacterium abscessus*; *Mav*: *Mycobacterium avium*; *Msm*: *Mycobacterium smegmatis*; *Mtb*: *Mycobacterium tuberculosis*; *Mbo*: *M. bovis*; *Mka*: *M. kansasii*

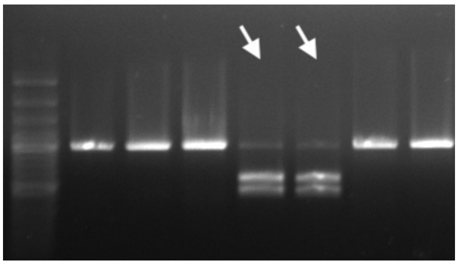

Figure S2. Screening of *M. smegmatis* recombinants for point mutations. Following transformation with the mixture of mutagenic oligonucleotides targeting both *dprE1* and *rpsL*, colonies isolated on streptomycin-containing were screened for the presence of point mutations in *dprE1*. A 944 bp fragment of *dprE1* was amplified by PCR, and then digested with *Kpn*2I. Wild-type *M. smegmatis* shows the undigested 959 bp product, while mixed populations (white arrows) show the undigested fragment and the two digestion products (525 and 419 bp). Left lane: Gene Ruler 100 bp Plus Ladder (ThermoFisher Scientific).

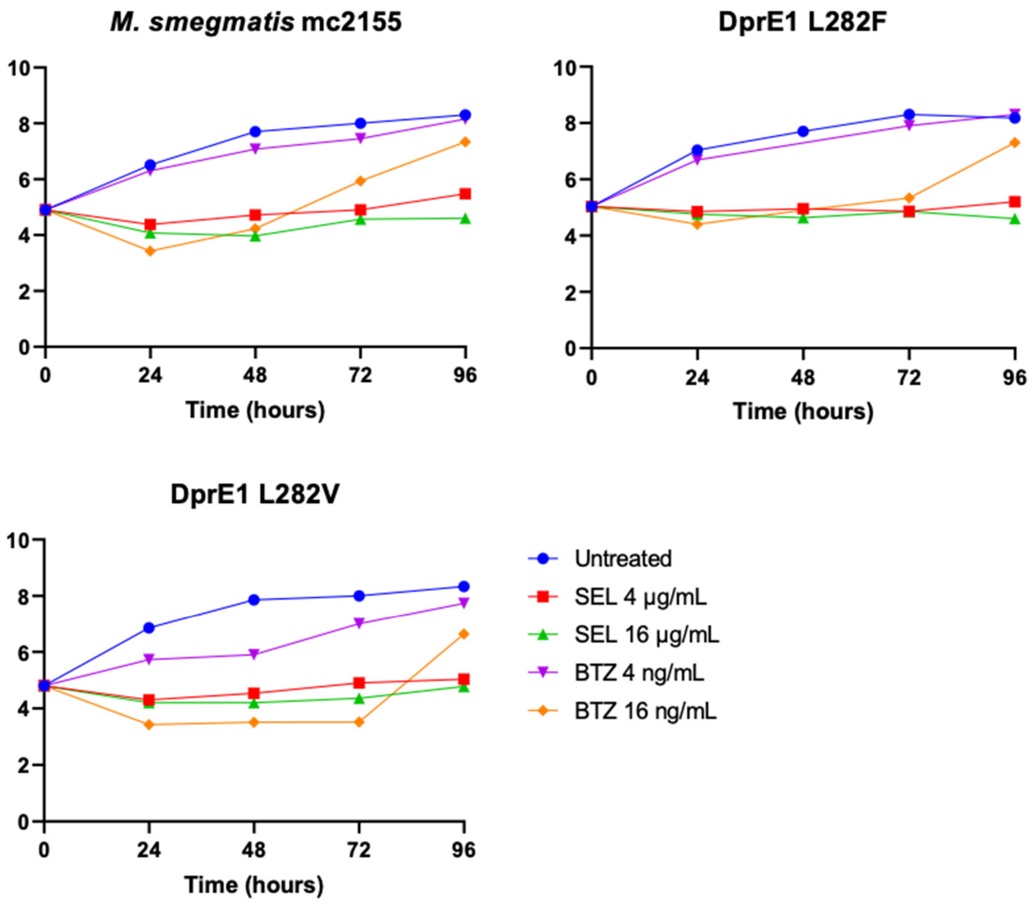

Figure S3. Time-kill kinetics of *M. smegmatis* DprE1 point mutants.
